# The S-layer Associated Serine Protease Homolog PrtX Impacts Cell Surface-Mediated Microbe-Host Interactions of *Lactobacillus acidophilus* NCFM

**DOI:** 10.3389/fmicb.2017.01185

**Published:** 2017-06-30

**Authors:** Brant R. Johnson, Sarah O’Flaherty, Yong Jun Goh, Ian Carroll, Rodolphe Barrangou, Todd R. Klaenhammer

**Affiliations:** ^1^Graduate Program in Microbiology, College of Agriculture and Life Sciences, North Carolina State University, RaleighNC, United States; ^2^Department of Food, Bioprocessing and Nutrition Sciences, North Carolina State University, RaleighNC, United States; ^3^Department of Medicine, Division of Gastroenterology and Hepatology, University of North Carolina at Chapel HillChapel Hill, NC, United States; ^4^Center for Gastrointestinal Biology and Disease, University of North Carolina at Chapel HillChapel Hill, NC, United States

**Keywords:** serine protease, S-layer, S-layer associated proteins, *Lactobacillus*, probiotic, intestinal barrier integrity, mucin, fibronectin

## Abstract

Health-promoting aspects attributed to probiotic microorganisms, including adhesion to intestinal epithelia and modulation of the host mucosal immune system, are mediated by proteins found on the bacterial cell surface. Notably, certain probiotic and commensal bacteria contain a surface (S-) layer as the outermost stratum of the cell wall. S-layers are non-covalently bound semi-porous, crystalline arrays of self-assembling, proteinaceous subunits called S-layer proteins (SLPs). Recent evidence has shown that multiple proteins are non-covalently co-localized within the S-layer, designated S-layer associated proteins (SLAPs). In *Lactobacillus acidophilus* NCFM, SLP and SLAPs have been implicated in both mucosal immunomodulation and adhesion to the host intestinal epithelium. In this study, a S-layer associated serine protease homolog, PrtX (*prtX*, *lba1578*), was deleted from the chromosome of *L. acidophilus* NCFM. Compared to the parent strain, the PrtX-deficient strain (Δ*prtX*) demonstrated increased autoaggregation, an altered cellular morphology, and pleiotropic increases in adhesion to mucin and fibronectin, *in vitro*. Furthermore, Δ*prtX* demonstrated increased *in vitro* immune stimulation of IL-6, IL-12, and IL-10 compared to wild-type, when exposed to mouse dendritic cells. Finally, *in vivo* colonization of germ-free mice with Δ*prtX* led to an increase in epithelial barrier integrity. The absence of PrtX within the exoproteome of a Δ*prtX* strain caused morphological changes, resulting in a pleiotropic increase of the organisms’ immunomodulatory properties and interactions with some intestinal epithelial cell components.

## Introduction

Lactic acid bacteria (LAB) are a clade of diverse Gram-positive, microaerophilic, and non-sporulating microbes which ferment hexoses primarily to lactic acid. Many of these bacteria, which include species from the genera *Lactococcus*, *Enterococcus*, *Pediococcus*, *Oenococcus*, *Streptococcus*, *Leuconostoc*, and *Lactobacillus*, have evolved through 1000s of years of fermentation in numerous food and drink substrates (Makarova et al., 2006; Douglas and Klaenhammer, 2010; Johnson and Klaenhammer, 2014). Predicated on this history of consumption in foods, many LAB are generally recognized as safe and serve vital industrial roles as starter and adjunct cultures for fermentation of dairy, vegetable, meat, and wine foodstuffs (Wood, 1998; Makarova et al., 2006).

In addition to their evolution to dairy environments, some LAB are niche-associated with the mucosal surfaces of animals, including the gastrointestinal and urogenital tracts (O’Flaherty and Klaenhammer, 2010a; Johnson and Klaenhammer, 2014). Many of these, most notably in the *Lactobacillus* genera, have been used as probiotics, which are defined by the FAO/WHO as “live microorganisms that, when administered in adequate amounts, confer a health benefit on the host” (FAO/WHO, 2002; Hill et al., 2014). One such organism is *Lactobacillus acidophilus* NCFM, an industrially significant probiotic strain, delivered commercially in various dairy and dietary supplement formulations for the past 35 years (Sanders and Klaenhammer, 2001). Furthermore, *L. acidophilus* NCFM is one of the most studied and well characterized probiotic bacteria (Sanders and Klaenhammer, 2001; Altermann et al., 2005; Klaenhammer et al., 2005, 2008).

Proteins and macromolecules at the cell surface of probiotic bacteria play a critical role in mediating strain-specific beneficial effects of probiotics toward the host (Lebeer et al., 2010; Bron et al., 2013). For *L. acidophilus* NCFM, the specificity of probiotic activity on the host has been characterized for numerous cell surface components, including lipoteichoic acid (Mohamadzadeh et al., 2011), sortase-dependent proteins (Call et al., 2015), an aggregation promoting factor (Goh and Klaenhammer, 2010), and a myosin-cross reactive protein (O’Flaherty and Klaenhammer, 2010b). Proteins at the Surface (S-) layer, which are non-covalently bound to the apical component of the cell wall peptidoglycan, are also of interest to the understanding of microbe-host interactions (Hynönen and Palva, 2013; Johnson and Klaenhammer, 2014).

Surface-layers are non-covalently bound, semi-porous, crystalline arrays comprised of self-assembling (glyco) protein subunits called S-layer proteins (SLPs; Sára and Sleytr, 2000). In *L. acidophilus*, the S-layer array is comprised of a dominant protein constituent, SlpA (46 kDa) with minor constituents SlpB (47 kDa) and SlpX (51 kDa; Goh et al., 2009). *In vitro* studies using intestinal epithelial cell lines suggest SLPs as a major factor in intestinal adhesion for *L. acidophilus* (Buck et al., 2005; Frece et al., 2005). Furthermore, SlpA of *L. acidophilus* NCFM has been shown to bind dendritic cell (DC) C-type lectin receptors (Konstantinov et al., 2008) and exert immunomodulatory signals which mitigate inflammatory disease states and promote maintenance of healthy intestinal barrier function (Lightfoot et al., 2015). Recent evidence has also shown that there are additional proteins which non-covalently co-localize to the outermost stratum of the cell surface with the S-layer, called S-layer associated proteins (SLAPs; Johnson et al., 2013, 2016). Due to the apparent importance of *Lactobacillus* cell surface proteins for probiotic roles, these SLAPs have been a prime target for investigation in recent years.

S-layer associated proteins were first characterized in *L. acidophilus* NCFM (Johnson et al., 2013), but have since been characterized in *Lactobacillus helveticus*, *Lactobacillus crispatus*, *Lactobacillus amylovorus*, and *Lactobacillus gallinarum* (Johnson et al., 2016). Notably, these SLAP-containing organisms are S-layer forming members of the *L. acidophilus*-*Lactobacillus delbrueckii* homology group. However, no SLAPs were proteomically identified within the non-covalent exoproteomes of the closely related, non-S-layer forming members of the homology group, including *Lactobacillus gasseri*, *Lactobacillus johnsonii*, as well as the taxonomic progenitor *L. delbrueckii* subsp. *bulgaricus* (Johnson et al., 2016). Preliminary functional analyses in *L. acidophilus* NCFM have revealed that SLAPs have a broad spectrum of both cellular and probiotic functionality, including cell division (Altermann et al., 2004; Johnson and Klaenhammer, 2016), autolysin activity (Johnson and Klaenhammer, 2016), immunomodulation (Johnson et al., 2013), and adhesion to extracellular matrices (Johnson and Klaenhammer, 2016; Hymes et al., 2016).

One of the most prevalent SLAPs identified in the exoproteome of *L. acidophilus* NCFM is a 72 kDa, uncharacterized serine protease encoded by the gene *lba1578* (Johnson et al., 2013). In this study, the S-layer associated serine protease, designated PrtX, was selected for functional analysis.

## Materials and Methods

### Bacterial Strains and Growth Conditions

Bacterial strains and plasmids used in this study are listed in **Table 1**. *L. acidophilus* strains were propagated in de Man Rogosa and Sharpe (MRS) broth (Difco) under ambient atmospheric conditions, statically or on MRS solid medium containing 1.5% (w/v) agar (Difco) under anaerobic conditions at 37°C, or at 42°C where indicated. Recombinant strains were selected in the presence of 2 μg/ml of erythromycin (Em; Sigma–Aldrich) and/or 2–5 μg/ml of chloramphenicol (Cm; Sigma). *Escherichia coli* strains were grown in brain heart infusion (Difco) medium at 37°C with aeration. *E. coli* EC101 was grown in the presence of 40 μg/ml kanamycin (Kn; Sigma–Aldrich) while NCK1911 and transformants were grown with 40 μg/ml Kn and 150 μg/ml Em. Counterselection of plasmid-free excision recombinants was performed using 5-fluorouracil-supplemented glucose semi-defined medium, as previously described (Goh et al., 2009).

**Table 1:** Strains, plasmids, and primers used in this study.

	Genotype or characteristics	Reference
***Lactobacillus acidophilus* strains**		
NCK56	Human intestinal isolate	Gilliland et al., 1975
NCK1909	NCK56 carrying a 315-bp in-frame deletion within the *upp* gene	Goh et al., 2009
NCK1910	NCK1909 harboring pTRK669; host for pORI-based counterselective integration vector	Goh et al., 2009
NCK2282	NCK1909 carrying a 1,966-bp deletion within the *lba1578* gene	This study
***Escherichia coli* strains**		
NCK1911	Host harboring pTRK935, Kn^r^, Em^r^	Goh et al., 2009
NCK2281	Host harboring pTRK1073, Kn^r^, Em^r^	This study
NCK1831	EC101 host for pORI-based plasmids	Law et al., 1995
**Plasmids**		
pTRK669	Ori (pWV01), Cm^r^, RepA^+^ thermosensitive	Russell and Klaenhammer, 2001
pTRK935	pORI upp-based counterselective integration vector, Em^r^	Goh et al., 2009
pTRK1073	pTRK935 with a mutated copy of *lba1578* cloned into BamHI/SacI site	This study
**Primers**		
1578BamHIF	GTAATAGGATCCTTTCCAGCCACATACTTTCT	This study
1578R	GTGACACCATCATTAAAGCA	This study
1578Soe	TTTAATGATGGTGTCACCAGTGGTACAACTTACATTGC	This study
1578SacIR	TAAAGTAGAGCTCCTCTGTAATTCCTGAACCAT	This study
1578up	TGGATGCAATTAGAGAAGGT	This study
1578dw	GGCATTAATCATTGCCTTAT	This study

*Restriction sites are underlined. Kn, Kanamycin; Em, Erythromycin; Cm, Chloramphenicol.*

### Molecular Techniques and Statistical Analysis

Genomic DNA from *L. acidophilus* strains was isolated using a Zymo Research Fungal/Bacterial DNA MiniPrep kit (Zymo Research). Plasmid DNA from *E. coli* was isolated using a QIAprep Spin Miniprep kit (Qiagen). Restriction enzyme digestions and ligations were performed using Roche restriction enzymes (Roche Diagnostics) and T4 DNA ligase (New England Biolabs), respectively. PCR primers (**Table 1**) were designed based on the genomic sequence data and synthesized by Integrated DNA Technologies (Coralville, IA, United States). PCRs were performed in Bio-Rad MyCycler thermocyclers (Bio-Rad Laboratories) using Choice-Taq Blue DNA polymerase (Denville Scientific) for screening of recombinants and *PfuUltra* II fusion HS DNA polymerase (Agilent Technologies) for cloning. PCR amplicons were analyzed on 0.8% agarose gels and purified using QIAquick Gel Extraction kits (Qiagen).

*Escherichia coli* EC101 cells were made competent using a rubidium chloride competent cell protocol (Hanahan, 1983). *L. acidophilus* cells were prepared for electrotransformation using a modified penicillin treatment protocol (Wei et al., 1995; Walker et al., 1996; Goh et al., 2009).

All statistical analyses were performed using unpaired student’s *t*-test. *P*-values below 0.05 were considered significant.

### Construction of a Δ*prtX* Strain of *L. acidophilus* NCFM

The *upp*-based counterselection gene replacement method (Goh et al., 2009) was used to create an internal deletion of 1966 bp in *prtX* (*lba1578*) of NCK1909, a *upp*-deficient background strain of *L. acidophilus* NCFM. Using splicing by overlap extension PCR (Horton et al., 1989), 439 and 548 bp regions flanking the deletion target were spliced together resulting in a 987 bp product with a BamHI restriction site added to the 5′ end and SacI restriction site to the 3′ end. This construct was digested with BamHI and SacI, then ligated into the polylinker of the similarly digested integration plasmid pTRK935 and transformed into competent *E. coli* EC101. The resulting recombinant plasmid, pTRK1073, was transformed into *L. acidophilus* NCK1909 harboring the helper plasmid pTRK669 (NCK1910). Single crossover integrants were screened as described previously (Goh et al., 2009). Colonies with the Δ*prtX* genotype were screened among the double recombinants recovered on glucose semi-defined medium agar plates containing 5-fluorouracil. Deletion was confirmed by PCR with primer pair 1578up and 1578dw (**Table 1**) and DNA sequencing. The resulting Δ*prtX* strain was designated NCK2282.

### Lithium Chloride Extraction of Extracellular S-Layer Associated Proteins

Non-covalently bound cell surface proteins, including SLPs and SLAPs were extracted from NCK1909 and Δ*prtX L. acidophilus* NCFM strains using LiCl denaturing salt, as described previously (Johnson et al., 2013). Proteins were quantified via bicinchoninic acid assay kit (Thermo Scientific) and visualized via SDS–PAGE using precast 4–20% Precise Tris-HEPES protein gels (Thermo Scientific). Gels were stained using AcquaStain (Bulldog Bio) according to the instructions of the manufacturer.

### Morphological Assessment and Electron Microscopy

Morphological assessment of *L. acidophilus* NCFM and Δ*prtX* was performed using a phase-contrast light microscope at 40× magnification (Nikon Eclipse E600). Cells were observed over a growth period of 24 h in MRS broth at 37°C. Pictures were taken using a QImaging MicroPublisher 5.0 RTV camera attachment at 1, 3, 5, 8, 10, and 13-h time points.

Sample processing for scanning electron microscopy (SEM) was performed by the Center for Electron Microscopy (CEM) at North Carolina State University. *L. acidophilus* NCFM strains were grown in 35 ml of MRS to logarithmic and stationary phases. Cells were pelleted by centrifugation at 3,166 × *g* for 15 min at room temperature. Pellets were resuspended in a fresh 1:1 (vol/vol) fixative mixture of 6% glutaraldehyde and 0.2 M sodium cacodylate (pH 5.5) and submitted to CEM for sample processing. SEM samples were viewed with a JEOL JSM 5900LV scanning electron microscope at 15 kV. For both light and electron microscopy analyses, images that were representative of the observed populations were used for assessment.

### Adherence Assays

Mucin and extracellular matrices (ECM) binding assays were performed as described previously (Goh and Klaenhammer, 2010). Mucin (type III from porcine stomach, Sigma) was dissolved in PBS to a final concentration of 10 mg/ml. Fibronectin (from human plasma, Sigma), collagen (type IV from human cell culture, Sigma), and laminin (from Engelbreth-Holm-Swarm murine sarcoma/basement membrane; Sigma) were diluted in 50 mM carbonate-bicarbonate buffer (pH 9.6, Sigma) to a final concentration of 10 μg/ml. For each assay, a Nunc Maxisorp 96-well microplate (Sigma) was coated with 100 μl/well of substrate and incubated at 4°C overnight. The wells were then washed twice with PBS (pH 7.4) to remove excess substrate before blocking with 150 μl of 2% bovine serum albumin (BSA) solution (Sigma) for 2 h at 37°C. Excess BSA was removed by two washes with PBS.

Bacterial cells were grown in MRS broth to stationary phase (16 h) in preparation for the assay. Cultures were centrifuged (1,771 × *g*, 15 min, room temperature), washed once with PBS, and resuspended in PBS (pH 4.75). Cell density was adjusted to ∼1 × 10^8^ CFU/mL based on previously calculated OD_600_/CFU ratios. Cell suspensions (100 μl) were added to each mucin or ECM-coated well. Initial cell counts of samples were enumerated on MRS plates. After incubation for 1 h at 37°C, the wells were gently washed five times with 200 μl/well of PBS. Adhered cells were recovered by adding 100 μl of 0.05% Triton X-100 solution (FisherBiotech, prepared in PBS) to each well and agitating on an orbital shaker (200 rpm) for 15 min. Cell suspensions were transferred into 900 μl of 0.1X MRS broth before being further diluted and plated in duplicate on MRS plates. Colonies were enumerated and calculated as a percent of relative adherence (mutant CFU/parent CFU), where parent (NCK1909) CFU were set at 100%.

### Bacterial-Dendritic Cell Co-incubation Assay and Cytokine Measurement

*In vitro* bacterial-DC co-incubation assays were performed as described previously (Johnson et al., 2013; Call et al., 2015). Cytokine measurements for IL-6, IL-10, and IL-12 were quantified using Single-Analyte ELISArray kits (Qiagen), according to the manufacturer’s instructions. Following cytokine quantification, the cytokine expression data were statistically compared between the parent and mutant strains using student *t*-test.

### Mono-Colonization of WT and Δ*prtX L. acidophilus* Strains in a Germ-Free Mouse Model

Germ-free 129S6/SvEv mice utilized for *in vivo* experiments were taken from breeding colonies maintained at the North Carolina State University Gnotobiotic Core of the Center for Gastrointestinal Biology and Disease, as described previously (Goh and Klaenhammer, 2014; Call et al., 2015). Mice were maintained in cages in germ-free flexible film isolators housed in a room with 12 h of light and darkness. They were provided access to a standard diet (Prolab RMH 3500, LabDiet) and water *ad libitum*. Germ-free status was evaluated at least once a month through culturing fecal samples in thioglycollate broth, blood agar, and Sabouraud agar. Prior to colonization with *L. acidophilus* strains, the mice were also verified germ-free by culturing fecal samples aerobically and anaerobically on plate count agar and MRS agar. Animal use protocols were approved by the Institutional Animal Care and Use Committee of North Carolina State University, Raleigh, NC, United States (Protocol 15-026-B). All mice handlers were certified by the American Association for Laboratory Animal Science. The Animal Welfare Assurance # is: A3331-01.

In preparation for mono-colonization of the germ-free mice, *L. acidophilus* NCK56 or the Δ*prtX* strain was propagated in MRS broth at 37°C overnight. Bacteria were harvested at stationary phase (16 h) via centrifugation (1735 × *g*, 10 min, room temperature). They were subsequently washed with and resuspended in PBS to an OD_600_ corresponding to 5 × 10^9^ CFU/ml for each strain. Germ-free 129S6/SvEv mice were separated into two experimental groups: the first treated with NCK56 (*n* = 6, 3 males and 3 females, 17–18 weeks old) and the second treated with Δ*prtX* (*n* = 6, 3 males and 3 females, 17–18 weeks old). Mice were gavaged with ∼1 × 10^9^ cells in 200 μl PBS per mouse. Fecal samples were collected from each mouse on the day of gavage (day 0), days 2, 5, and 7. Fecal samples were weighed, resuspended in 1 ml of PBS, diluted and plated on MRS agar for enumeration.

### *In Vivo* FITC-Dextran Epithelial Barrier Integrity in a Germ-Free Mouse Model

Germ-free 129S6/SvEv mice were mono-colonized with either wild-type (WT) or Δ*prtX* strains of *L. acidophilus* NCFM, as described above. On the day of the assay, mice were denied access to food but allowed access to water *ad libitum* for 3 h, after which 150 μl of 3–5 kDa fluorescein isothiocyanate (FITC)-dextran at a concentration of 80 mg/ml was administered to each mouse using an oral gavage needle. Two hours after administration of the FITC-dextran, blood was collected using a 1 ml tuberculin syringe (BD) and stored in Microtainer serum separator tubes (BD). Blood was placed in the dark for 1 h and allowed to clot, after which the serum was separated via centrifugation (13,000 rpm, 4°C). Fluorescence in the serum was measured using a FLOUStar Optima Microtiter plate reader (BMG Technologies) with the excitation filter set for 490 nm, the emission filter set for 520 nm, and a 1250 gain setting for fluorescence intensity. FITC-dextran fluorescence was measured in the serum collected from NCK56 mono-colonized mice (*n* = 5, 2 males, 3 females, 17–18 weeks old) and Δ*prtX* mono-colonized mice (*n* = 5, 2 males, 3 females, 17–18 weeks old). Serum from a single germ-free control was used to measure background fluorescence. Using a standard of known concentrations of FITC-dextran in PBS, the concentration of FITC-dextran was calculated.

## Results

### PrtX, a S-Layer Associated Serine Protease of *L. acidophilus*

One of the most prevalent SLAPs in the non-covalently bound exoproteome of *L. acidophilus* NCFM is a 72 kDa uncharacterized serine protease encoded by the gene *lba1578* (Johnson et al., 2013). The protein is 684 amino acids in length and contains a predicted signal peptide sequence located between amino acid 34 and 35 (VKA-AD). *In silico* analysis of LBA1578 revealed that there is discreet homology to serine proteases in *Lactobacillus acetotolerans* DSM 20749 (33% identity), *Lactobacillus gigeriorum* DSM 23908 (32%), *Lactobacillus pasteurii* DSM 23907 (31%), *L. amylovorus* DSM 20351 (31%), *Lactobacillus kitasatonis* DSM 16761 (31%), and *Lactobacillus amylolyticus* DSM 11664 (30%). Due to the uncharacterized nature of LBA1578 and the conserved annotation of serine protease among discreet homologs, this protein was designated PrtX.

**FIGURE 1:**
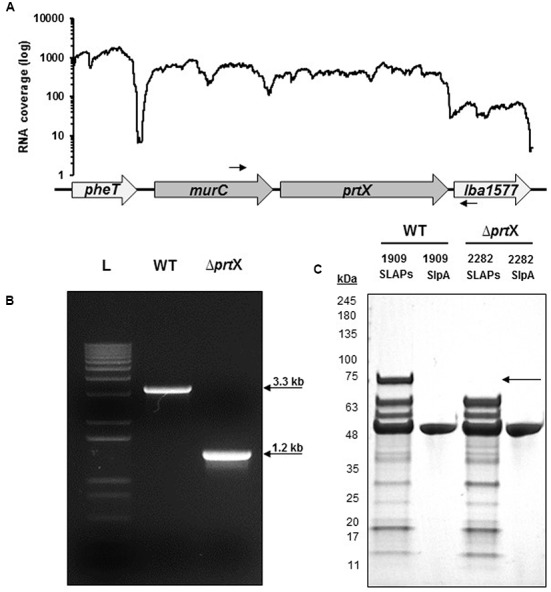
The gene encoding PrtX was deleted from the chromosome of *Lactobacillus acidophilus* NCFM. **(A)** RNA-seq analysis demonstrates that *prtX* is polycistronically expressed with *murC*, which encodes for a UDP-*N*-acetylmuramate-L-alanine ligase putatively involved in peptidoglycan biosynthesis. Black arrows indicate the forward and reverse primer pair used to confirm the deletion of *prtX*. **(B)** Gel electrophoresis of PCR products using the primers indicated in **(A)** for the parent strain (WT; 3.3 kb) compared to the PrtX-deficient strain (Δ*prtX*; 1.2 kb). The deletion was confirmed by sequencing. **(C)** SDS–PAGE of the non-covalently bound extracellular S-layer proteins (SLP) and S-layer associated proteins (SLAPs) isolated from both WT and Δ*prtX*. Absence of the 72 kDa band corresponding to PrtX within the SLAP extracts from Δ*prtX* confirmed the elimination of PrtX from the exoproteome of Δ*prtX*.

Using RNA-seq transcriptional analysis from a previous study (Johnson et al., 2016), it was determined that *lba1578* (*prtX*) mRNA is polycistronically expressed downstream of *murC*, which encodes a UDP-*N*-acetylmuramate-L-alanine ligase putatively involved in peptidoglycan biosynthesis (**Figure 1A**). Because PrtX is an uncharacterized serine protease which is prominently featured in the non-covalent exoproteome in *L. acidophilus*, it was selected for functional analysis. The *upp*-based counterselective gene replacement method (Goh et al., 2009) was used to create a markerless chromosomal deletion of 1966 base pairs in the coding region of *prtX* in *L. acidophilus* NCFM (**Figure 1B**). SDS–PAGE analysis of extracted SLAPs confirmed the presence of PrtX in the non-covalently bound exoproteome of the WT *L. acidophilus* NCFM and its absence from the Δ*prtX* strain (**Figure 1C**).

### Growth and Morphology of Δ*prtX*

**FIGURE 2:**
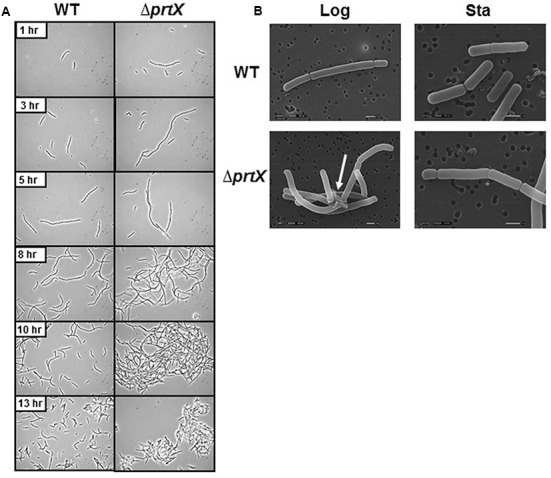
**(A)** The cellular morphology of the wild-type (WT) and mutant (Δ*prtX*) strains were assessed using phase-contrast light microscopy over a 13-h growth period. **(B)** Scanning electron micrographs were taken of WT and Δ*prtX* at logarithmic and stationary phases.

The cellular morphology of the Δ*prtX* was examined using light microscopy over a 24-h period of growth in MRS broth (**Figure 2A**). Compared to the parent strain (WT), the Δ*prtX* strain demonstrated aberrant morphology and increased autoaggregation phenotypes (**Figure 2A**). Specifically, during lag phase (13 h; OD_600_ 0.1–0.2) the Δ*prtX* cells appeared to have a longer chain length compared to WT cells. Logarithmic phase (5–10 h; OD_600_ 0.5–1.0) cell chains of the Δ*prtX* mutant were only similarly longer than WT. Furthermore, the Δ*prtX* cells presented an increased autoaggregation phenotype which was not observed in the WT strain (**Figure 2A**). By stationary phase (13 h; OD_600_ 1.4; **Figure 2A**) the autoaggregation phenotype was still observed in the Δ*prtX* cells.

For a more detailed observation of these morphological phenotypes, WT and Δ*prtX* strains were examined using SEM at logarithmic phase (OD_600_ 0.6) and stationary phase (OD_600_ 1.9; **Figure 2B**). The SEM micrographs mirror much of the morphological assessment with the light microscope. Notably, protein complexes were repeatedly observed in the micrographs of log phase Δ*prtX* cells at 8,500× magnification, suggesting a build-up of proteins at the cell surface due to poor protein turnover (**Figure 2B**). Such complexes were not observed in the WT sample (**Figure 2B**). Furthermore, these complexes were not observed in the stationary phase samples of Δ*prtX or* WT cells. However, it is notable that compared to the WT stationary phase sample, much fewer cells were available for observation under the microscope. This is perhaps due to the difficulty of fixing large clusters of cells onto the platform for SEM. Collectively, these morphological data implicate that PrtX may be involved in protein turnover at the surface during log phase cell division.

### Adherence of Δ*prtX* to Mucin and Extracellular Matrices

**FIGURE 3:**
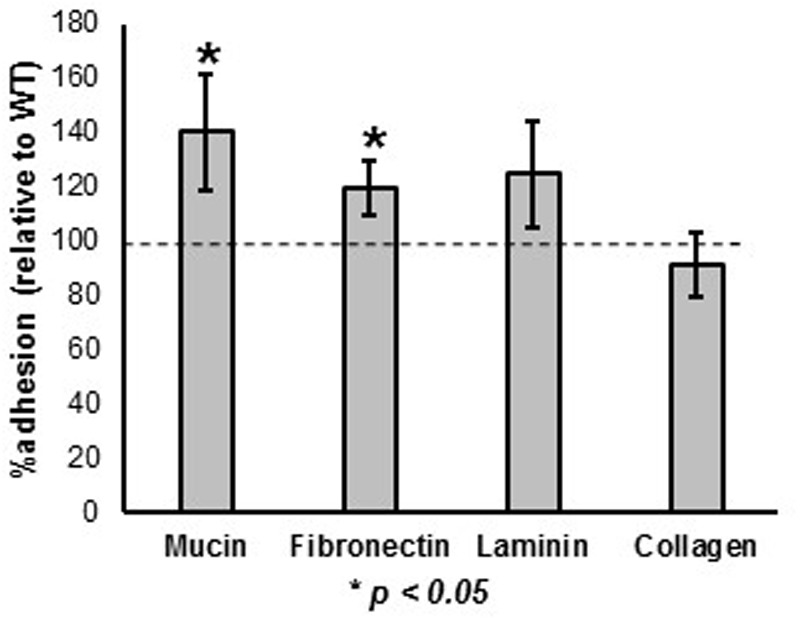
The ability of the Δ*prtX* mutant to bind to mucin and extracellular matrices (ECM) was assessed. Compared the WT reference (dotted line), Δ*prtX* showed a significant increase in binding to mucin and fibronectin. Asterisks indicate statistical significance (*p* < 0.001). Adherence assays were performed in triplicate; all error bars represent confidence intervals.

The ability of the *prtX* mutant to bind to mucin and the ECM fibronectin, collagen, and laminin was assessed and compared to WT, *in vitro* (**Figure 3**). The Δ*prtX* strain demonstrated a 40% increase in binding capacity to mucin, compared to the parent strain (**Figure 3**, *p* < 0.001). Similarly, Δ*prtX* showed a 20% increase in binding to fibronectin (**Figure 3**, *p* < 0.001). While Δ*prtX* appeared to have an increased binding capacity for laminin by 25%, this increase was not statistically significant. Finally, relative to WT, the Δ*prtX* strain did not show any increase in binding to collagen (**Figure 3**).

### *In Vitro* Immunomodulation of Mouse DC Cells Exposed to Δ*prtX*

**FIGURE 4:**
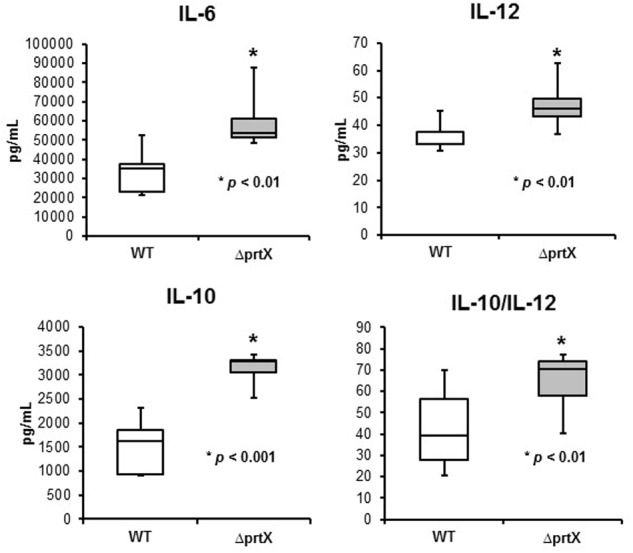
The immunomodulatory phenotype of Δ*prtX* (gray) compared to WT (white) was measured using a murine dendritic cell co-incubation assay. Cytokines IL-6, IL-12, and IL-10 were assessed using enzyme-linked immunosorbent assays. For all cytokines measured, Δ*prtX* demonstrated a significant increase in immunomodulation compared to WT (IL-6, *p* < 0.01; IL-12, *p* < 0.006; IL-10, *p* < 0.001). The IL-10/IL-12 ratio was also significantly increased in Δ*prtX* (*p* < 0.01). Co-incubation assays were performed in duplicate or triplicate; bars on the box-whisker plots represent the range.

The immunomodulatory potential of the Δ*prtX* strain was assessed using an *in vitro* bacterial/murine DC co-incubation assay. Relative to the WT strain, Δ*prtX* demonstrated an overall increase in immunostimulation of the cytokines IL-6, IL-12, and IL-10 (**Figure 4**). Specifically, Δ*prtX* induced production of the pro-inflammatory cytokine IL-6 in DC at twice the level of that of the parent strain (**Figure 4**, IL-6). The pro-inflammatory cytokine IL-12 was also induced in Δ*prtX* compared to WT in a less pronounced, but statistically significant manner (**Figure 4**, IL-12). Similar to IL-6, in Δ*prtX* induced DC, the anti-inflammatory cytokine IL-10 was produced at twice the level of that produced by the WT-induced DC (**Figure 4**, IL-10). Although Δ*prtX* induced both the pro-inflammatory cytokine IL-12 and the anti-inflammatory cytokine IL-10, the IL-10/IL-12 ratio, which is a measure of the balance between pro-inflammatory and anti-inflammatory states (O’Garra and Murphy, 2009; Hamer et al., 2010), was higher in Δ*prtX* than in WT (**Figure 4**, IL-10/IL-12).

### *In Vivo* Mono-Colonization and Epithelial Barrier Integrity of Germ-Free Mice

**FIGURE 5:**
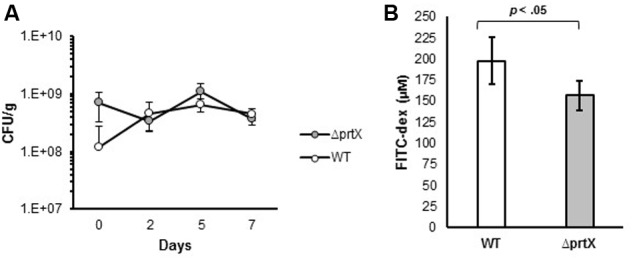
*In vivo* assessment of germ-free mice colonized with either WT or Δ*prtX* strains of *L. acidophilus* NCFM. **(A)** Mice were monocolonized with WT (white, *n* = 6) or Δ*prtX* (gray, *n* = 6) over 7 days and colonization levels were assessed by plating fecal samples on MRS agar. **(B)** Total gastrointestinal epithelial barrier integrity was assessed through the measurement of FITC-dextran in the serum of mice previously fed FITC-dex. Mice colonized with Δ*prtX* had a statistically significant reduction in FITC-dex in the serum, indicating an increase in epithelial barrier integrity (*p* < 0.05).

Due to the increased adhesion and immunomodulatory properties of Δ*prtX*, *in vitro*, the biological relevance of the Δ*prtX* strain was explored in an *in vivo* germ-free mouse model. Germ-free mice were colonized with ∼1 × 10^9^ CFU/g of either WT or Δ*prtX* strains; this mono-colonization was measured over 7 days (**Figure 5A**). Both WT and Δ*prtX* strains colonized the germ-free mice at similar rates and by day 7 the bacteria had colonized at 4.56 × 10^8^ and 3.71 × 10^8^ CFU/g fecal samples for WT and Δ*prtX*, respectively (**Figure 5A**). The gastrointestinal epithelial barrier integrity was examined in a second set of mice (**Figure 5B**). These mice were similarly colonized with WT or Δ*prtX* strains and fed FITC-dextran; the resulting FITC-dextran in the blood serum correlated to the gastrointestinal barrier integrity of the mouse. Compared to WT, serum from the Δ*prtX* colonized mice demonstrated a 21% reduction in FITC-dextran levels (**Figure 5B**, *p* < 0.05). These data indicate that the intestinal epithelial barrier integrity was improved in mice colonized with Δ*prtX* relative to mice colonized with WT.

## Discussion

In this study, the serine protease designated PrtX, was functionally and phenotypically characterized. Following the creation of a clean chromosomal deletion of *prtX*, the corresponding mutant strain (Δ*prtX*) was assessed for cellular morphology, *in vitro* mucin and ECM adhesion, *in vitro* immunomodulation, and *in vivo* mouse colonization and intestinal epithelial barrier integrity.

Morphological assessment of the Δ*prtX* isogenic mutant revealed a visible increase in autoaggregation, compared to the parent strain. This increased autoaggregation phenotype was further evaluated using SEM, in which logarithmic Δ*prtX* cells were observed to present aberrant cellular topology. These observations suggest that PrtX may be involved with protein turnover at the cell surface of *L. acidophilus* NCFM. Gene deletion studies of serine proteases in other *Lactobacillus* species, including PrtB in *L. delbrueckii* subsp. *bulgaricus* and PrtH in *L. helveticus*, have not reported a similar morphological or autoaggregation phenotypes (Gilbert et al., 1996; Pederson et al., 1999; Genay et al., 2009; Sadat-Mekmene et al., 2011). However, this difference in observed phenotypes may be due to the subcellular localization of the referenced serine proteases. PrtB and PrtH are cell-envelope serine proteases which are cell-membrane attached, whereas *prtX* is non-covalently attached to the cell wall peptidoglycan. Due to the lack of homologs for PrtX, it is difficult to contextualize the results of this study with similar studies in *Lactobacillus* species. However, a similar increased autoaggregation phenotype was reported during the functional analysis of S-layer associated autolysin, AcmB, in *L. acidophilus* NCFM (Johnson and Klaenhammer, 2016). Future proteomic analysis of the proteomic complexes observed in Δ*prtX* will undoubtedly aid in the description of PrtX and the proteins it may process.

Proteins localized at the cell surface of *L. acidophilus* NCFM are important mediators of adhesion to host intestinal epithelial mucus layer and ECM, *in vitro* (Buck et al., 2005; Azcarate-Peril et al., 2009; Goh and Klaenhammer, 2010; O’Flaherty and Klaenhammer, 2010b; Call et al., 2015; Hymes et al., 2016; Johnson and Klaenhammer, 2016). In this study, the Δ*prtX* strain demonstrated a significant increase in binding to mucin and fibronectin. These results are consistent with a recent analysis of the serine protease, PrtS in *Streptococcus thermophilus*, in which a PrtS-deficient strain bound to Caco-2 epithelial cells twice as effectively as the parent strain (Kebouchi et al., 2016). Furthermore, previous analysis of the S-layer associated fibronectin binding protein, FpbB, in *L. acidophilus* NCFM revealed that FpbB has specificity for binding fibronectin and mucin, *in vitro* (Hymes et al., 2016). Though the exact mechanism remains unclear, it is possible that the PrtX-deficient strain is deficient in protein turnover at the cell surface, including SLAPs such as FpbB, resulting in increased binding to mucin and fibronectin, specifically. It is possible that the generalized increase in autoaggregation resulted in an inflation of the CFU counts upon plating. However, this was not observed across all epithelial cell components (e.g., collagen), lending credence to the results’ representation of true adherence. More in-depth quantitative proteomic studies could elucidate this mechanism even further.

In addition to their role in adhesion, cell surface proteins of *L. acidophilus* NCFM, including SLP and SLAPs, have also been examined for roles in immunomodulation (Konstantinov et al., 2008; Mohamadzadeh et al., 2011; Johnson et al., 2013; Call et al., 2015; Lightfoot et al., 2015). Previous research regarding immunomodulation and CEPs, specifically, has focused on the production of bioactive compounds released during casein proteolysis in milk (Matar et al., 2001; Silva and Malcata, 2005; Korhonen and Pihlanto, 2006; Shah, 2007). For example, milk fermented with a non-proteolytic variant of *L. helveticus* was found to have a suppressed mucosal immune response compared to milk fermented by the WT strain of *L. helveticus* (Matar et al., 2001). Recent evidence, however, has pointed to a more specific immunomodulatory role of serine proteases in *Lactobacillus*. An extracellular lactocepin serine protease in *Lactobacillus paracasei* was found to exert anti-inflammatory effects by selectively degrading pro-inflammatory chemokines, such as CXCL10 (von Schillde et al., 2012). In the present study, we found that the absence of PrtX in the Δ*prtX* mutant resulted in a generalized increase in DC expression of cytokines IL-6, IL-12, and IL-10, compared to WT. One cytokine induced in DCs exposed to Δ*prtX* was the anti-inflammatory IL-10, which has been proposed as a biological therapy for chronic irritable bowel disease (IBD), including Crohn’s disease and colitis (Li and He, 2004). It is possible that PrtX may directly degrade certain cytokines, which may explain the observed accumulation of cytokines when DCs were exposed the PrtX-deficient strain, though this direct mechanism remains to be discovered.

Previous work has been performed in *L. acidophilus* NCFM using an *in vivo* germ-free mouse model. Through mutational analysis, sortase, an enzyme which covalently couples extracellular proteins containing an LPXTG motif to the cell surface, was found to contribute to gut retention of *L. acidophilus* NCFM (Call et al., 2015). Similarly, the glycogen biosynthesis pathway in *L. acidophilus* NCFM contributes to gut fitness and retention, *in vivo* (Goh and Klaenhammer, 2014). In the present study, the biological relevance of PrtX was examined in germ-free mice. Specifically, intestinal epithelial barrier integrity was assessed in germ-free mice colonized with either Δ*prtX* or the WT strains. Dysbiosis of the normal enteric gastrointestinal microbiome has been demonstrated as a key factor in the initiation and amplification of IBD (Tamboli et al., 2004). Recent evidence has implicated enteric and commensal proteases as one such mechanism for pathogenesis in IBD (Pruteanu et al., 2011; Carroll and Maharshak, 2013). In *Enterococcus faecalis*, a Gram-positive commensal bacterium of the gastrointestinal tract, extracellular proteases have been shown to mediate intestinal epithelial barrier disruption and contribute to intestinal inflammation (Steck et al., 2011; Maharshak et al., 2015). Here, we show that the Δ*prtX* mutant causes increased epithelial barrier integrity in the germ-free mice compared to WT colonized mice. PrtX may directly interact with various ECM components of intestinal epithelia eliciting a direct effect on intestinal epithelial integrity. It is also possible that the indirect immunomodulatory effects of Δ*prtX* in the germ-free mouse model resulted in the increased barrier integrity, as cytokines can modulate tight junction structure and function in intestinal epithelial cells (Walsh et al., 2000).

## Conclusion

We have demonstrated that PrtX is a cell surface, S-layer associated serine protease homolog. Deletion of *prtX* from the chromosome of *L. acidophilus* NCFM revealed a distinct alteration in cell morphology, autoaggregation, and increased cell binding to mucin and fibronectin. Expression of IL-6, IL-12, and IL-10 was increased, *in vitro*, in mouse DCs. Furthermore, colonization of the Δ*prtX* strain in a germ-free mouse model improved gastrointestinal epithelial barrier integrity. Collectively, these data indicate that PrtX acts on the intestinal cell matrix and likely is involved in protein turnover during microbial cell division. PtrX, therefore, likely impacts how proteins are displayed within the microbial cell surface matrix and may alter the structure and properties of the epithelial intestinal cell matrix.

## Author Contributions

BJ designed the study, conducted the research, interpreted the results, and wrote the paper. SOF, YG, and IC conducted the research, interpreted the results, and contributed/edited the paper. RB interpreted results and contributed/edited the paper. TK designed the study, interpreted results, and contributed/edited the paper.

## Conflict of Interest Statement

The authors declare that the research was conducted in the absence of any commercial or financial relationships that could be construed as a potential conflict of interest.
